# Comprehensive radiologic-pathologic correlation in systemic sclerosis-associated interstitial lung disease: identification of an early-stage CT findings

**DOI:** 10.1007/s11604-025-01922-2

**Published:** 2025-12-18

**Authors:** Taiki Fukuda, Yasuhiko Yamano, Kaori Ishida, Tomonori Tanaka, Ryoko Egashira, Hiromitsu Sumikawa, Mikiko Hashisako, Junya Tominaga, Mai Matsumura, Midori Ueno, Daisuke Yamada, Yuki Ko, Yusei Nakamura, Hiroya Ojiri, Hiroto Hatabu, Reoto Takei, Kensuke Kataoka, Tomoki Kimura, Yasuhiro Kondoh, Junya Fukuoka, Takeshi Johkoh

**Affiliations:** 1 Center for Pulmonary Functional Imaging, Department of Radiology, Brigham and Women’s Hospital and Harvard Medical School, 75 Francis St, Boston, MA 02115 USA; 2 Department of Radiology, The Jikei University School of Medicine, 3-25-8 Nishi-Shinbashi, Minato-ku, Tokyo, 105-8461 Japan; 3 Department of Respiratory Medicine and Allergology, Aichi Medical University, 1-1 Iwasaku Karimata, Nagakute, Aichi 480-1195 Japan; 4 Department of Respiratory Medicine and Allergy, Tosei General Hospital, 160 Nishioiwakecho, Seto, Aichi 489-8642 Japan; 5 Department of Pathology and Laboratory Medicine, Kansai Medical University, 2-5-1 Shinmachi, Hirakata, Osaka 573-1010 Japan; 6 Department of Pathology, Kobe University Hospital, 7-5-2 Kusunoki-cho, Chuo-ku, Kobe, Hyogo 650-0017 Japan; 7 Department of Radiology, Faculty of Medicine, Saga University, 5-1-1 Nabeshima, Saga-City, Saga 849-0937 Japan; 8 Satsuma Lab, Hawkes Institute, University College London, 1St Floor, 90 High Holborn, London, WC1V 6BH UK; 9 Department of Radiology, NHO Kinki-Chuo Chest Medical Center, 1180 Nagasone-cho, Kita-ku, Sakai-City, Osaka 591-8555 Japan; 10 Department of Pathology, Kyushu University Hospital, 3-1-1 Maidashi, Higashi-ku, Fukuoka-City, Fukuoka 812-8582 Japan; 11 Department of Diagnostic Radiology, Tohoku University Hospital, 1-1 Seiryo-machi, Aoba-ku, Sendai, Miyagi 980-8574 Japan; 12 Department of Pathology, Saitama Medical University, 38 Morohongo, Moroyamamachi, Iruma-Gun, Saitama, 350-0495 Japan; 13 Diagnostic and Interventional Radiology, Faculty of Medicine, The University of Osaka Graduate School of Medicine, 2-1 Yamada-oka, Suita, Osaka 565-0871 Japan; 14 Department of Clinical Radiology, Graduate School of Medical Sciences, Kyushu University, 3-1-1 Maidashi, Higashi-ku, Fukuoka-City, Fukuoka 812-8582 Japan; 15 Department of Pathology Informatics, Nagasaki University Graduate School of Biomedical Sciences, 1-7-1 Sakamoto, Nagasaki-City, Nagasaki 852-8501 Japan; 16 Department of Radiology, Kansai Rosai Hospital, 3-1-69 Inabaso, Amagasaki, Hyogo 660-8511 Japan

**Keywords:** Systemic sclerosis, Interstitial lung disease, High-resolution computed tomography, Radiologic-pathologic correlation, Pulmonary fibrosis

## Abstract

**Purpose:**

To perform comprehensive radiological-pathological correlation in systemic sclerosis-associated interstitial lung disease (SSc-ILD) and identify characteristic findings, including subtle abnormalities potentially representing early-stage CT findings.

**Material and methods:**

This retrospective study included 28 SSc-ILD patients who underwent surgical lung biopsy between July 2008 and July 2018. Two chest radiologists independently reviewed whole-lung high-resolution CT (HRCT) images, with the other two radiologists evaluating biopsy sites. Faint amorphous nodular opacity (FANO) was defined as a small, faint nodular opacity superimposed on amorphous ground-glass opacity (GGO) within 1 cm of the pleural surface, showing a band-like distribution parallel to the pleura. Three pulmonary pathologists performed histological evaluation. Discrepancies were resolved through consensus, with CT-pathologic correlation established through joint radiologist-pathologist review.

**Results:**

Twenty-eight patients (mean age, 57 years ± 10; 15 men) were evaluated with 79 biopsy specimens. Nonspecific interstitial pneumonia was the predominant pattern on whole-lung HRCT (21 patients, 75%) and pathology (17 patients, 61%). At biopsy sites, GGO was most frequent (92%), followed by reticulation (84%). Reticulation was accompanied by GGO in nearly all cases, reflecting underlying diffuse fibrotic changes. Reticulation patterns with or without traction bronchiolectasis corresponded to varying fibrosis types, spatial distribution, and architectural destruction severity. Specifically, irregular reticulation with traction bronchiolectasis indicated dense fibrosis with severe destruction, representing UIP-like features. FANO was observed in 18 patients (64%), predominantly in anterolateral upper lobes, and corresponded pathologically to perivenular fibrosis and peribronchiolar metaplasia with or without mucostasis. Longitudinal evaluation (median 32.5 months) in 14 patients showed progression in 71%; half of these showed coalescence into subpleural curvilinear opacities with reticulation.

**Conclusion:**

SSc-ILD demonstrates predominantly diffuse fibrotic changes. Irregular reticulation with traction bronchiolectasis indicates UIP-like features, potentially identifying patients at risk for progression. FANO, observed most commonly in anterolateral upper lobes, frequently progresses to reticulation on longitudinal follow-up, suggesting potential value for early-stage detection.

**Secondary abstract:**

SSc-ILD demonstrated diffuse fibrotic changes as a characteristic feature, while irregular reticulation with traction bronchiolectasis indicated UIP-like fibrosis with severe architectural destruction. FANO, a newly described finding observed in 64% of cases, was predominantly in anterolateral upper lobes and corresponded to perivenular fibrosis and peribronchiolar metaplasia, representing potential early-stage changes.

**Supplementary Information:**

The online version contains supplementary material available at 10.1007/s11604-025-01922-2.

## Introduction

Systemic sclerosis (SSc) is an autoimmune disease characterized by endothelial dysfunction and fibrosis resulting from excessive collagen production by fibroblasts [1]. Pulmonary involvement is common in patients with SSc, and interstitial lung disease (ILD) represents the most frequent manifestation, occurring in approximately 50–65% of patients on high-resolution CT (HRCT) imaging [2–4]. The characteristic CT findings in systemic sclerosis-associated interstitial lung disease (SSc-ILD) are well established, including bilateral lower lobes and peribronchovascular-predominant ground-glass opacity (GGO) and reticulation, accompanied by traction bronchiectasis and decreased lung volume [5, 6]. Nonspecific interstitial pneumonia (NSIP) is most frequently observed, with some studies reporting NSIP features on HRCT in more than 80% of cases [7–11].

HRCT represents the most valuable and sensitive tool for analyzing pulmonary lesions in SSc [12]. Although numerous reports indicate that NSIP is the predominant pattern in SSc-ILD, significant clinical heterogeneity exists among these patients. The clinical course of ILD in SSc patients varies considerably: some remain stable without treatment, while others experience progressive disease [12]. Given this clinical heterogeneity, a detailed correlation between imaging and pathological findings is essential for a comprehensive understanding and stratification of disease progression.

However, lung biopsies in SSc-ILD patients are typically performed only when there is a mismatch between clinical symptoms and HRCT findings, or when clinicians need to exclude other diseases [13]. This practice limits the number of lung biopsies performed. To the best of our knowledge, only four articles have provided comprehensive radiologic-pathologic correlation in SSc-ILD [8, 14–16], constraining our understanding of the disease spectrum.

Given these limitations, there is an unmet need for CT-pathologic correlation studies in SSc-ILD. CT findings of SSc-ILD demonstrate lower lobe predominance in severity and extent [5, 6], with upper lobes showing less severe involvement. Based on our clinical experience and supported by previous observations of disease progression patterns [17], these subtle upper lobe CT findings may represent early-stage manifestations of SSc-ILD. Understanding the pathological basis of CT findings is particularly important given recent advances in targeted therapies for SSc-ILD [18]. In this context, identifying early-stage changes and progression patterns may provide valuable insights into disease mechanisms and guide treatment optimization.

Therefore, we aimed to perform a comprehensive CT-pathological correlation in SSc-ILD to elucidate the pathological basis of various CT findings, including detailed characterization of fibrotic patterns and identification of subtle abnormalities that might represent early-stage manifestations.

## Materials and methods

### Study design

This retrospective study was approved by the institutional review board (34–142[11293]) with waiver of informed consent. Between July 2008 and July 2018, 69 patients were diagnosed with SSc-ILD at our institution, of whom 28 underwent surgical lung biopsy (SLB) and were included in this study.

**Fig. 1:**
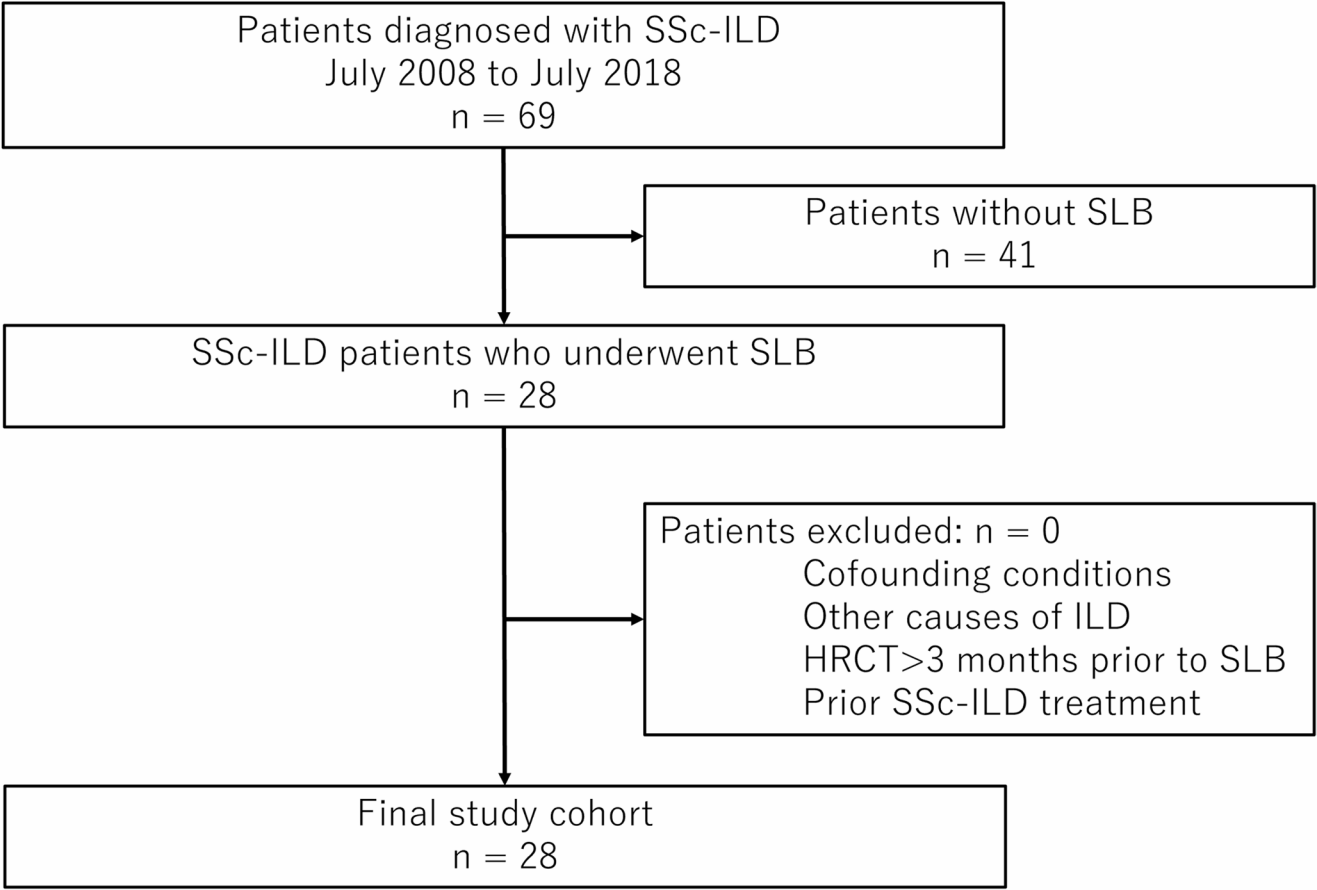
Patient selection flowchart. *HRCT* high-resolution CT, *ILD* interstitial lung disease, *SLB* surgical lung biopsy, *SSc-ILD* systemic sclerosis-associated interstitial lung disease

The inclusion criteria were a diagnosis of SSc according to the 2013 American College of Rheumatology/European League Against Rheumatism classification criteria [19] and histologically confirmed ILD by SLB. Exclusion criteria were confounding conditions such as infection or congestive heart failure, other causes of ILD, HRCT obtained more than three months prior to SLB, and patients who had received any specific treatment for SSc-ILD prior to CT examinations or pathological specimen collection. Patient demographics, smoking history, and pulmonary function test results were obtained from electronic medical records. The flowchart of the patient selection process is illustrated in Fig. 1.

### Radiologic examination and image analysis

CT images were reconstructed with contiguous 0.5-mm axial thickness using lung window settings on inspiration (width = 1500 Hounsfield unit [HU]; level = −600 HU). The reconstruction kernel was FC52, and the reconstruction algorithms used were either filtered back projection (FBP), adaptive iterative dose reduction (AIDR), or AIDR 3D. Deep learning reconstruction was not used. All CT images were obtained using a routine clinical protocol on a 16-slice and 80-slice multi–detector row CT (MDCT) scanner (Aquilion 16, Aquilion PRIME, Canon Medical Systems Corporation, Otawara, Japan).

**Fig. 2:**
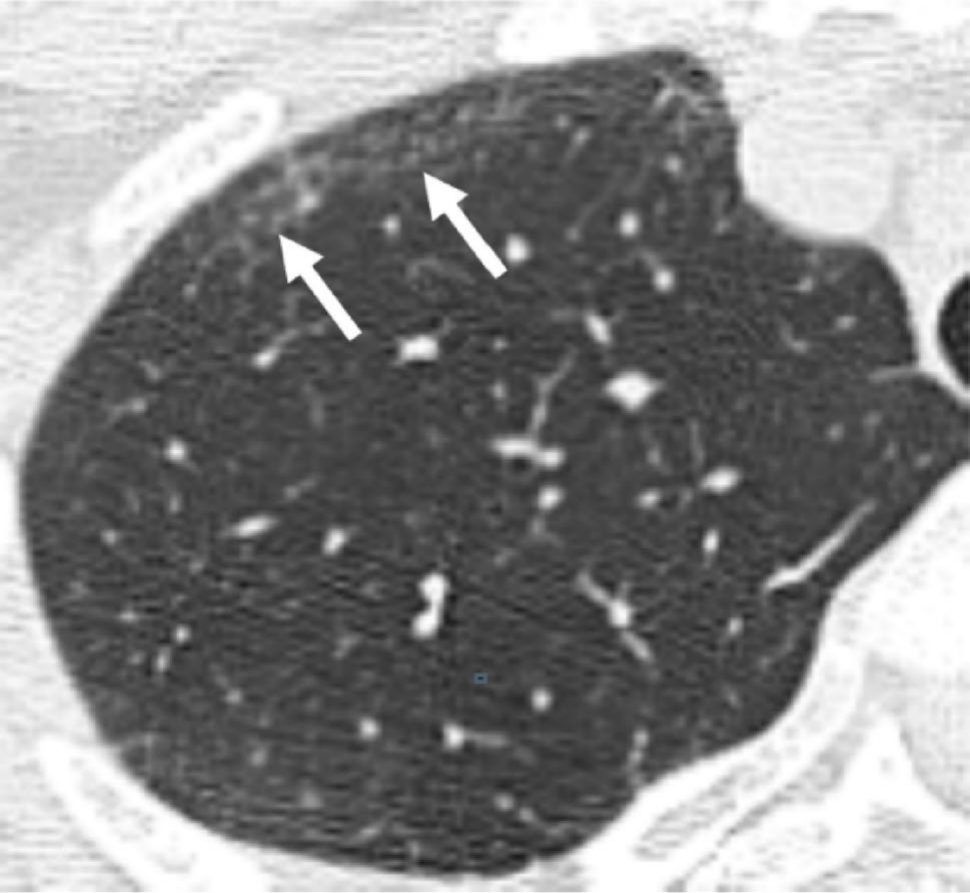
A 39-year-old woman with FANO. Unenhanced axial chest HRCT shows small, faint nodules on amorphous ground-glass opacity within 1 cm of the pleura, with a band-like distribution parallel to the pleura. The finding is particularly prominent along the anterolateral portions (arrows). *FANO* faint amorphous nodular opacity, *HRCT* high-resolution CT

Whole-lung HRCT was independently reviewed by two chest radiologists with 21 years (R.E.) and 22 years (H.S.) of experience in HRCT interpretation, who were blinded to the patients’ clinical and pathological data to evaluate both the main CT patterns and specific CT findings. Discrepancies between observers were discussed until consensus was reached. CT patterns were classified according to idiopathic interstitial pneumonias (IIPs) criteria [20]. We defined NSIP with organizing pneumonia (NSIP + OP) as NSIP features with prominent consolidation [21–23] and evaluated bronchiolocentric interstitial pneumonia (BIP) based on features described in the literature [24]. Eight HRCT findings were assessed: GGO, consolidation, reticulation, centrilobular nodules, traction bronchiectasis, emphysema, honeycombing, and faint amorphous nodular opacity (FANO). HRCT findings were interpreted per Fleischner Society guidelines [25, 26]. FANO was defined as small, faint nodules on amorphous GGO within 1 cm of the pleural surface, showing a band-like distribution parallel to the pleura (Fig. 2). FANO distribution was analyzed by the same two radiologists through consensus by lobar division (upper vs. middle/lower lobes) and axial division (anterior one-third vs. posterior two-thirds of the lung field). The association between FANO and the main CT patterns was analyzed. Longitudinal changes in FANO were qualitatively evaluated in patients who underwent follow-up CT scans at ≥ 12 months after the pre-biopsy CT, focusing on disease progression or stability.

For CT-pathologic correlation, lung areas corresponding to biopsy sites were extracted and independently reviewed by two chest radiologists with 35 years (T.J.) and 14 years (T.F.) of experience in HRCT interpretation, blinded to clinical and pathological data. Any discrepancies were finalized through mutual agreement. Seven HRCT findings were evaluated: GGO, consolidation, irregular reticulation with traction bronchiolectasis, fine reticulation with traction bronchiolectasis, fine reticulation without traction bronchiolectasis, emphysema, and FANO. Irregular reticulation was defined as a coarse and clear linear shadow, while fine reticulation was defined as a fine and obscure interlacing line shadow.

### Pathologic examination

Histological diagnosis was based on specimens obtained via video-assisted thoracoscopic surgery. Every specimen was diagnosed by three experienced pulmonary pathologists with 15 years (T.T.), 11 years (M.H.), and 8 years (K.I.) of experience in ILD, who were blinded to the patients’ clinical and radiological data. Discrepancies between pathologists were discussed until consensus was reached. Parenchymal abnormalities were evaluated and categorized according to the current classification of IIPs [20]. Additionally, we defined NSIP + OP as a distinct histopathological pattern, corresponding to the radiologic pattern. If different histological diagnoses were made between several biopsy sites in the same case, a histological pattern of usual interstitial pneumonia (UIP) in any lobe was classified as having UIP [27].

All specimens underwent hematoxylin–eosin and Elastic van Gieson staining. Pathological evaluation focused on fibrotic and inflammatory changes, emphysematous changes, and specific features such as perivenular fibrosis and peribronchiolar metaplasia. Histological findings for each specimen were correlated with the corresponding CT findings based on side-by-side analysis.

### Precise CT-pathologic correlation

The resection site on the pre-biopsy CT was identified by referring to the resection scar on the post-biopsy CT. CT findings at biopsy sites and corresponding pathological findings were evaluated to determine CT-pathologic correlations. Correlation analysis was performed by three chest radiologists with 35 years (T.J.), 21 years (R.E.), and 14 years (T.F.) of experience in HRCT interpretation and three thoracic pathologists with 15 years (T.T.), 11 years (M.H.), and 8 years (K.I.) of experience in ILD. Final correlation decisions were made through consensus between the radiologists and pathologists. Additionally, we compared detection rates of emphysematous changes between CT and pathological examination.

### Statistical analysis

Continuous variables were expressed as mean ± standard deviation or median with interquartile range (IQR) as appropriate, and categorical variables as numbers with percentages. The interobserver agreement was classified as follows: poor (κ = 0–0.20), fair (κ = 0.21–0.40), moderate (κ = 0.41–0.60), good (κ = 0.61–0.80), and excellent (κ = 0.81–1.00) [28]. Kappa values were calculated for CT findings at whole-lung HRCT and biopsy sites. All statistical analyses were performed using R software version 4.5.0 (R Foundation for Statistical Computing, Vienna, Austria).

## Results

### Patient characteristics

Our study cohort of 28 patients had a mean age of 57 ± 10 years (range, 34–71 years). Fifteen patients were male (54%) and 13 were female (46%), with 21 patients (75%) having a history of smoking (Table 1). Complete data were available for all variables, including patient demographics, smoking history, pulmonary function test results, CT findings, and pathological findings.

**Table 1:** Patient demographics and characteristics

Characteristics	Value
Total patients	28
*Age, years*	
Mean ± SD	57 ± 10
Range	34–71
*Sex*	
Male	15 (54)
Female	13 (46)
Smoking history	21 (75)
*Pulmonary function tests*	
FVC, L	2.5 ± 0.8
FVC, % predicted	83.4 ± 18.1
FEV_1_, L	2.1 ± 0.6
FEV_1_, % predicted	84.1 ± 18.9
FEV_1_/FVC_,_ %	83.5 ± 7.5
DLco, % predicted	52.5 ± 17.6

Continuous data are presented as mean ± standard deviation (SD). Categorical data are presented as numbers of patients, with percentages in parentheses

*DLco* diffusing capacity of the lungs for carbon monoxide, *FEV₁* forced expiratory volume in one second, *FVC* forced vital capacity

### HRCT and pathological patterns in SSc-ILD

A total of 79 biopsy specimens were examined from the 28 patients. HRCT and pathological patterns in SSc-ILD patients are summarized in Table 2. NSIP was the predominant pattern across both diagnostic modalities, observed in 21 patients (75%) on HRCT and 17 patients (61%) on pathological examination. UIP was the second most common pattern, identified more frequently on pathological examination (9 patients, 32%) than on HRCT (3 patients, 11%). NSIP + OP was observed in three patients (11%) on HRCT and two patients (7.1%) on pathological examination, while BIP was observed in one case (3.6%) on HRCT.

**Table 2:** HRCT and pathological patterns in SSc-ILD patients

	HRCT pattern (n = 28)	Pathological pattern (n = 28)
NSIP	21 (75)	17 (61)
UIP	3 (11)	9 (32)
NSIP + OP	3 (11)	2 (7.1)
BIP	1 (3.6)	0 (0)

Categorical data are presented as numbers of findings, with percentages in parentheses

*BIP* bronchiolocentric interstitial pneumonia, *HRCT* high-resolution CT, *NSIP* nonspecific interstitial pneumonia, *NSIP* + *OP* nonspecific interstitial pneumonia with organizing pneumonia, *OP* organizing pneumonia, *SSc-ILD* systemic sclerosis-associated interstitial lung disease, *UIP,* usual interstitial pneumonia

### Whole-lung HRCT findings

The results are shown in Table 3. GGO, reticulation, and traction bronchiectasis were observed in all 28 patients. Interobserver agreement for whole-lung HRCT findings showed moderate to excellent agreement (Supplemental Table 1).

**Table 3:** Whole-lung HRCT findings in patients with SSc-ILD

HRCT findings	Value (n = 28)
Ground-glass opacity	28 (100)
Consolidation	5 (18)
Reticulation	28 (100)
Centrilobular nodules	10 (36)
Traction bronchiectasis	28 (100)
Emphysema	17 (61)
Honeycombing	1 (3.6)
FANO	18 (64)

Categorical data are presented as numbers of findings, with percentages in parentheses. *FANO* faint amorphous nodular opacity, *HRCT* high-resolution CT, *SSc-ILD* systemic sclerosis-associated interstitial lung disease

### FANO characteristics and longitudinal evolution

**Fig. 3:**
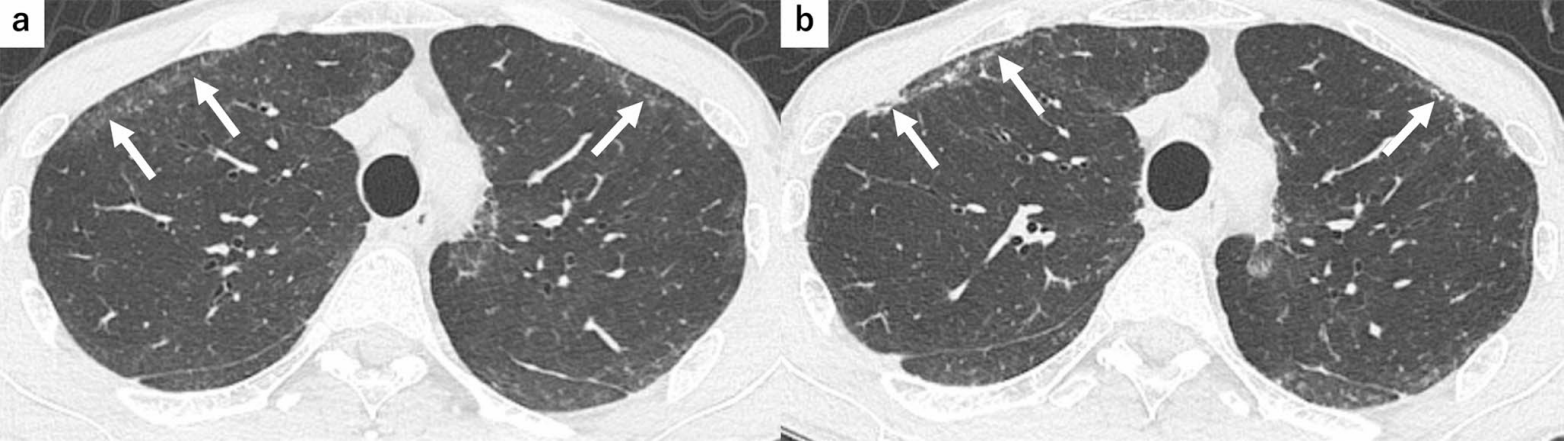
A 57-year-old man with FANO. **a** Pre-biopsy unenhanced axial chest HRCT shows FANO in the bilateral anterior upper lobes (arrows). **b** Follow-up HRCT at 2 years demonstrates progression with coalescence into subpleural curvilinear opacities and reticulation (arrows). *FANO* faint amorphous nodular opacity, *HRCT* high-resolution CT

FANO was observed in 18 patients (64%), all involving the anterior portions of the upper lobes with anterolateral distribution (Fig. 2). Additional distribution was noted in upper lobe posterior (4 patients, 22%), middle/lower lobe anterior (6 patients, 33%), and middle/lower lobe posterior (3 patients, 17%). Among the 18 patients with FANO, the majority showed NSIP or NSIP-related patterns: NSIP in 14 patients (78%) and NSIP + OP in two patients (11%) (Table 4). Of the 18 patients with FANO, follow-up CT scans obtained ≥ 12 months after the pre-biopsy CT were available in 14 patients (median follow-up: 32.5 months; IQR: 23.0–48.9 months). Longitudinal evaluation revealed progression in 10 patients (71%). Among these, 5 patients showed moderate progression with reticulation, volume loss, and coalescence of scattered FANO, forming thin, curvilinear opacities parallel to the pleural surface. The remaining 5 patients showed subtle progression with a slight increase in the extent of FANO. Four patients (29%) showed minimal or no change. A representative case showing the progression of FANO is presented in Fig. 3.

**Table 4:** Distribution of FANO on HRCT according to location and associated CT patterns

Category	Value (n = 18)
*Anatomical distribution*	
Upper lobe, anterior	18 (100)
Upper lobe, posterior	4 (22)
Middle/lower lobe, anterior	6 (33)
Middle/lower lobe, posterior	3 (17)
*CT patterns associated with FANO*	
NSIP	14 (78)
NSIP + OP	2 (11)
UIP	1 (5.6)
BIP	1 (5.6)

Categorical data are presented as numbers of findings, with percentages in parentheses

*BIP* bronchiolocentric interstitial pneumonia, *FANO* faint amorphous nodular opacity, *HRCT* high-resolution CT, *NSIP* nonspecific interstitial pneumonia, *NSIP* + *OP* nonspecific interstitial pneumonia with organizing pneumonia, *UIP* usual interstitial pneumonia

### CT-pathologic correlation

Table 5 summarizes the HRCT findings at biopsy sites on prebiopsy CT images and their correlation with pathology. Interobserver agreement for CT findings at biopsy sites demonstrated moderate to good agreement (Supplemental Table 2). Among the 79 biopsy sites of the prebiopsy CT images, the most frequent HRCT features were GGO in 73 sites (92%). Reticulation of any type was present in 66 sites (84%), followed by emphysema in 18 sites (23%), and consolidation in 7 sites (8.9%).

**Table 5:** Correlation between HRCT findings and pathological features at biopsy sites

HRCT findings	n = 79	Pathological findings
Ground-glass opacity	73 (92)	Inflammatory cellular infiltration, Edema (intra-alveolar, interstitial), Peribronchiolar metaplasia with or without mucostasis, focal subacute change, Diffuse fibrous thickening of alveolar septa
Consolidation	7 (8.9)	Organizing pneumonia with or without fibrosis, edema (intra-alveolar, interstitial)
Reticulation	66 (84)	
Irregular reticulation with traction bronchiolectasis	48 (61)	Dense fibrosis with severe architectural destruction
Fine reticulation with traction bronchiolectasis	16 (20)	Dense/ loose fibrosis with mild to moderate architectural destruction
Fine reticulation without traction bronchiolectasis	2 (2.5)	Mild alveolar fibrosis without architectural destruction
Emphysema	18 (23)	Emphysematous change
FANO	6 (7.6)	Perivenular fibrosis, Peribronchiolar metaplasia with or without mucostasis

Categorical data are presented as numbers of findings, with percentages in parentheses

*FANO* faint amorphous nodular opacity, *HRCT* high-resolution CT

GGO on HRCT corresponded histopathologically to both fibrotic findings (diffuse fibrous thickening of alveolar septa) and non-fibrotic findings, such as inflammatory cellular infiltration and edema.

**Fig. 4:**
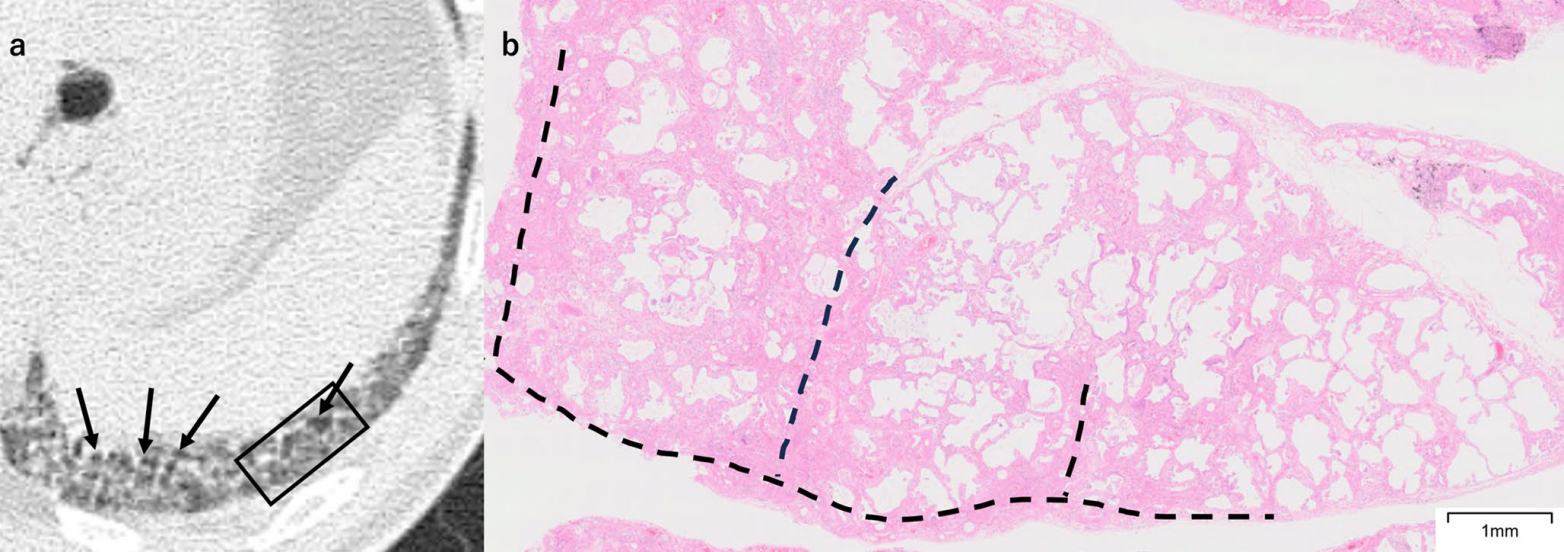
A 52-year-old man with irregular reticulation with traction bronchiolectasis. **a** Unenhanced axial chest HRCT shows irregular reticulation of varying thickness with traction bronchiolectasis (arrows) accompanied by surrounding ground-glass opacity. The SLB resection site is indicated by a black square. **b** The SLB specimen shows diffuse fibrosis with fibrotic thickening of the alveolar septa. Although diffuse, the fibrosis is more prominent near the interlobular septa (dashed line). *HRCT* high-resolution CT, *SLB* surgical lung biopsy

**Fig. 5:**
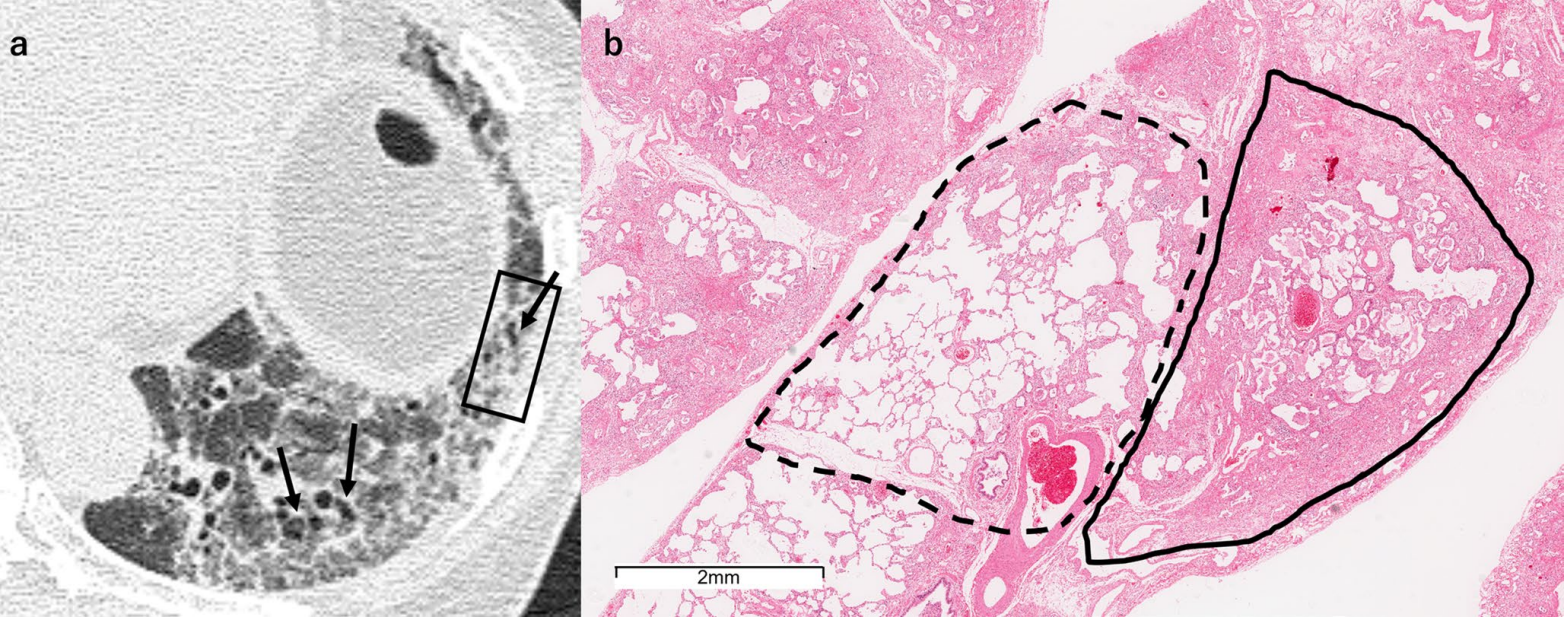
A 64-year-old woman with irregular reticulation with traction bronchiolectasis. **a** Unenhanced axial chest HRCT shows irregular reticulation of varying thickness with traction bronchiolectasis (arrows) accompanied by surrounding ground-glass opacity. The SLB resection site is indicated by a black square. **b** The SLB specimen shows diffuse alveolar septal fibrosis with varying severity from lobule to lobule. Solid lines indicate lobules with severe fibrosis, and dashed lines indicate lobules with mild fibrosis. *HRCT* high-resolution CT, *SLB* surgical lung biopsy

**Fig. 6:**
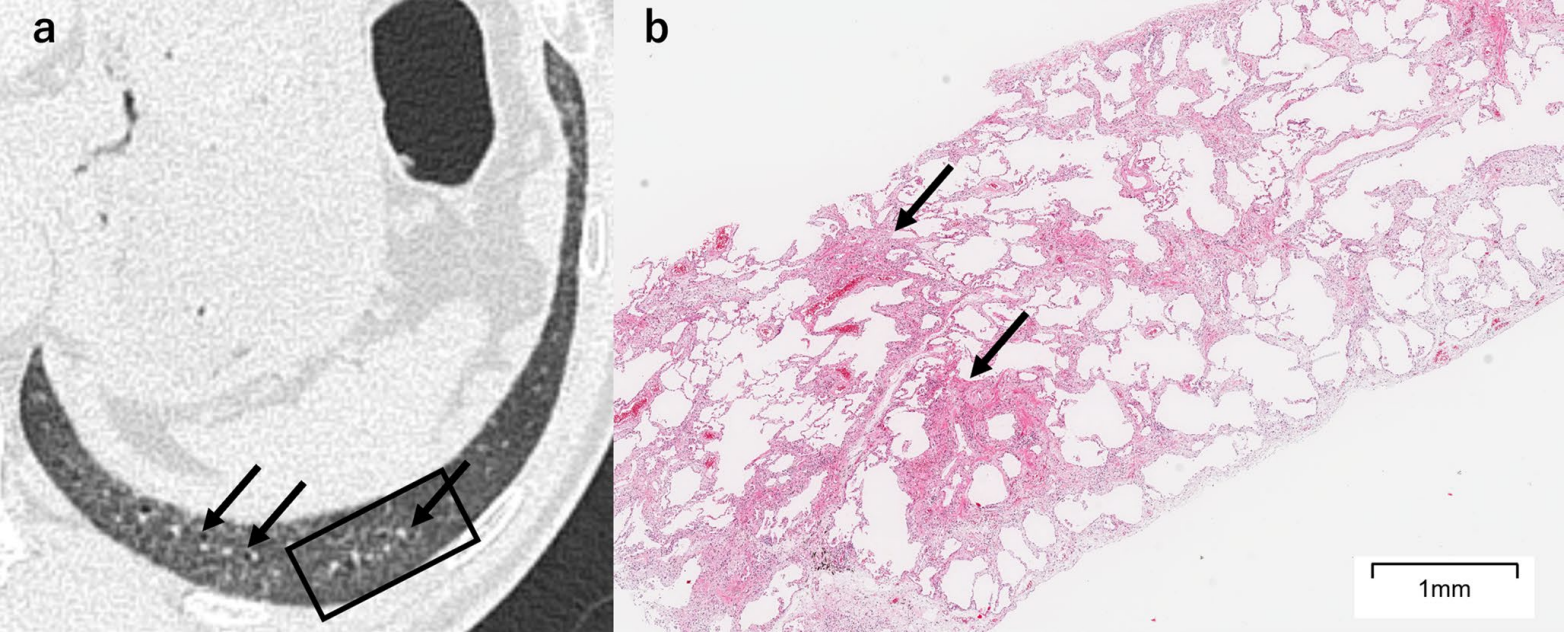
A 56-year-old man with fine reticulation with traction bronchiolectasis. **a** Unenhanced axial chest HRCT shows fine reticulation with traction bronchiolectasis (arrows), accompanied by surrounding ground-glass opacity. The SLB resection site is indicated by a black square. **b** The SLB specimen shows diffuse alveolar septal fibrosis, predominantly consisting of loose fibrosis, though focal areas of dense fibrosis are also present (arrows). *HRCT* high-resolution CT, *SLB* surgical lung biopsy

**Fig. 7:**
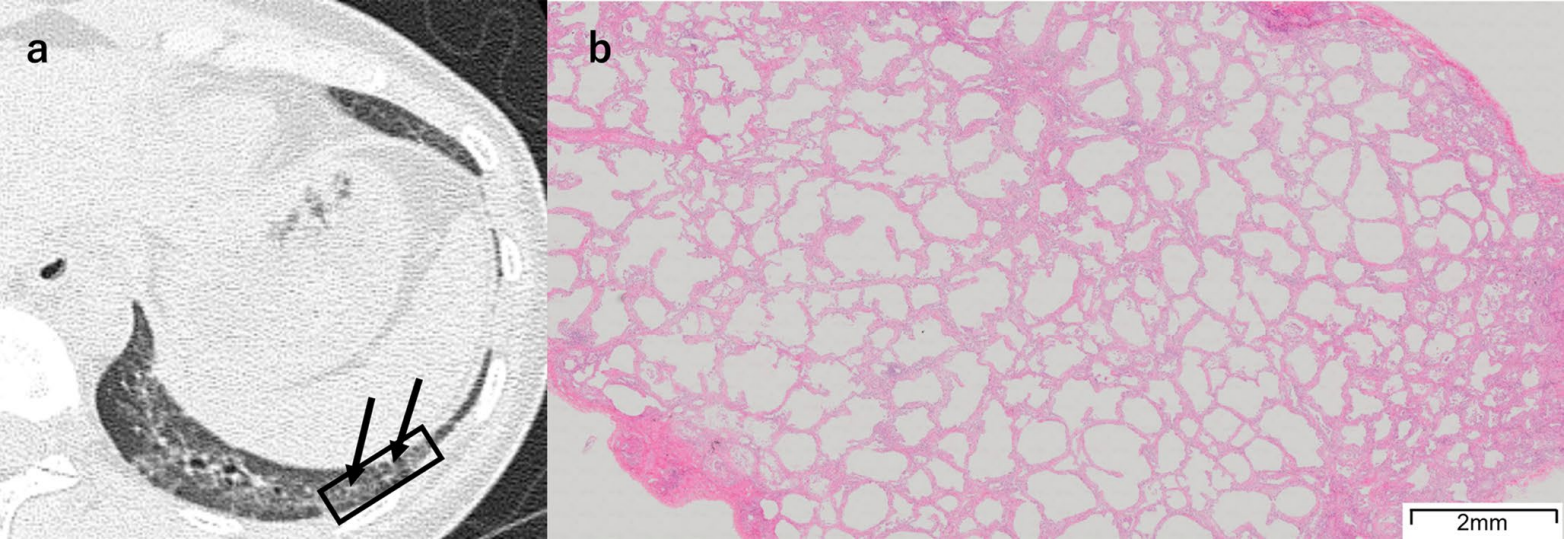
A 39-year-old woman with fine reticulation without traction bronchiolectasis. **a** Unenhanced axial chest HRCT shows fine reticulation, accompanied by surrounding ground-glass opacity (arrows). The SLB resection site is indicated by a black square. **b** The SLB specimen shows diffuse and homogeneous alveolar septal fibrosis. *HRCT* high-resolution CT, *SLB* surgical lung biopsy

Reticulation corresponded to fibrosis and was accompanied by architectural destruction in cases in which HRCT showed traction bronchiolectasis. Cases with irregular reticulation with traction bronchiolectasis (48 sites, 61%) exhibited denser fibrosis with more heterogeneous distribution of fibrosis both within lobules and between different lobules (Figs. 4, 5) compared to those with fine reticulation with traction bronchiolectasis (16 sites, 20%) (Fig. 6). Additionally, the irregular reticulation cases showed severe architectural destruction, while the fine reticulation pattern showed mild to moderate architectural destruction. In contrast, fine reticulation without traction bronchiolectasis was found in only 2 sites (2.5%), corresponding to mild alveolar fibrosis without architectural destruction (Fig. 7). All cases with reticulation showed some degree of GGO, except for one case that involved three biopsy sites. These accompanying GGO consistently demonstrated diffuse alveolar septal fibrosis.

Consolidation was histologically observed as organizing pneumonia, with and without fibrosis. Additionally, it corresponded to intra-alveolar and interstitial edema.

**Fig. 8:**
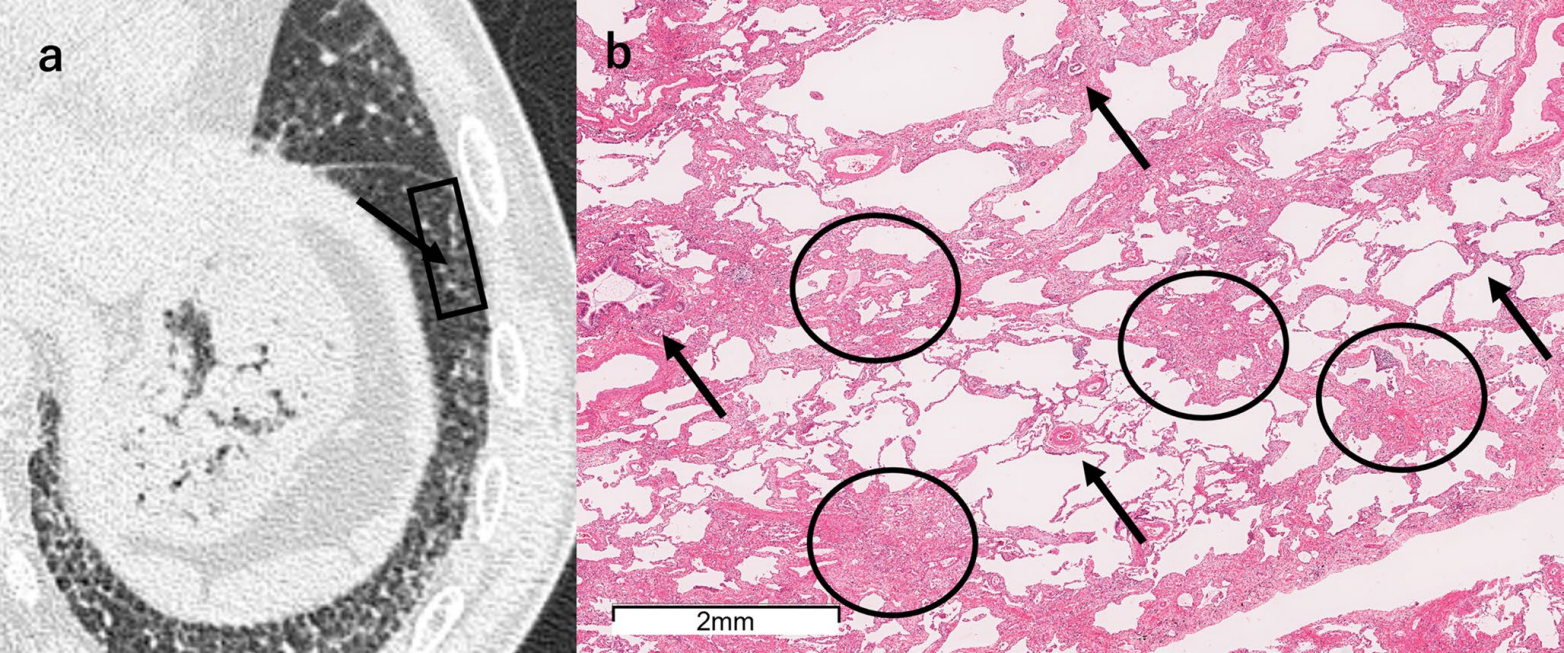
A 61-year-old man with FANO. **a** Unenhanced axial chest HRCT shows small, faint nodules superimposed on amorphous ground-glass opacity in the left lower lobe (arrow). The SLB resection site is indicated by a black square. **b** The SLB specimen shows fibrosis (circles) located in areas neither perilobular nor adjacent to small airways (arrows), consistent with perivenular fibrosis. *FANO* faint amorphous nodular opacity, *HRCT* high-resolution CT, *SLB* surgical lung biopsy

FANO (6 sites, 7.6%) corresponded to either scattered fibrotic lesions characterized by perivenular fibrosis or peribronchiolar metaplasia with or without mucostasis (Fig. 8, Supplemental Fig. 1).

### Emphysematous changes and smoking history

Prebiopsy CT images revealed emphysema in 18 biopsy sites (23%) from nine patients. In contrast, histopathological emphysema was identified in 45 specimens (57%) from 18 patients. Among the 18 patients with histopathological emphysema, 14 had a smoking history and four were never-smokers (Supplemental Fig. 2).

## Discussion

We performed comprehensive radiological-pathological correlation in SSc-ILD to investigate characteristic CT findings and their underlying pathological features. NSIP was the predominant pattern on both HRCT (75%) and pathology (61%), with UIP being the second most common. While diffuse fibrosis was characteristic, reticulation type with or without traction bronchiolectasis reflected fibrosis severity with varying degrees of spatial heterogeneity and architectural destruction. Notably, we newly describe FANO as a potential early-stage CT finding predominantly in the anterolateral upper lobes, corresponding to perivenular fibrosis and peribronchiolar metaplasia, likely representing early-stage disease manifestations.

The predominance of NSIP in SSc-ILD is consistent with previous studies, with reported frequencies of 76–90% on HRCT and 64–100% on pathological examination [7–11]. UIP is typically the second most common pattern, reported in 9.5–12.5% on HRCT [9, 10] and 0–15% pathologically [7, 9–11]. Our pathological diagnosis identified more UIP cases than whole-lung CT, likely due to imaging-histological discrepancy [29]. NSIP + OP may correspond to previously reported centrilobular fibrosis with predominant bronchocentric distribution [16]. Other less common patterns have been reported, including organizing pneumonia, diffuse alveolar damage, pleuroparenchymal fibroelastosis [7, 9, 11], but were not observed in our cohort.

Reticulation patterns and presence of traction bronchiolectasis on HRCT reflected underlying fibrosis characteristics in SSc-ILD. Almost all lesions with reticulation were accompanied by GGO, which consistently showed diffuse fibrous thickening of alveolar septa, reflecting characteristic NSIP-like fibrosis with uniform distribution and relatively preserved architecture compared to UIP [30]. Importantly, almost all reticulation was accompanied by traction bronchiolectasis. Among cases with traction bronchiolectasis, those with irregular reticulation exhibited denser fibrosis with more heterogeneous distribution and more severe architectural destruction compared to fine reticulation cases, indicating UIP-like fibrotic features. Indeed, an overlap of UIP has been reported in histopathological NSIP in ILD patients [31], supporting our observations. In contrast, the few cases of fine reticulation without traction bronchiolectasis corresponded to less severe fibrotic change showing homogeneous alveolar septal fibrosis, representing the uniform fibrotic pattern characteristic of NSIP [20].

Identifying UIP-like fibrotic features extends beyond diagnostic classification. Recent evidence suggests that focal UIP-like fibrosis comprising ≥ 10% of disease area serves as a key prognostic factor in progressive pulmonary fibrosis, regardless of underlying etiology [32]. Furthermore, although the optimal timing for initiating antifibrotic therapy in SSc-ILD remains unclear, nintedanib has been shown to reduce forced vital capacity decline in SSc-ILD patients in the SENSCIS trial [33]. Therefore, identification of UIP-like features may help identify SSc-ILD patients at risk for progressive disease and optimize antifibrotic treatment timing in future therapeutic strategies.

FANO represents a potential early-stage CT finding in SSc-ILD, observed in 64% of cases predominantly in the anterolateral upper lobes. Pathologically, FANO corresponded to perivenular fibrosis and peribronchiolar metaplasia with or without mucostasis, comprising both fibrotic and non-fibrotic components. These pathological findings did not correspond to any specific diagnostic pattern. This aligns with previous studies showing that subtle fibrotic findings corresponding to interstitial lung abnormalities may not meet the pathological criteria for established patterns [34].

The combination of these subtle pathological changes with their predominant location in the less severely affected upper lobes, contrasting with the characteristic lower lobe predominance of SSc-ILD [5, 6], supports the interpretation of FANO as an early-stage manifestation. This interpretation is further reinforced by Launay et al. [17], who described SSc-ILD progression from posterior to anterior and from lower to upper lung zones. In patients with early-stage SSc-ILD, FANO might theoretically be the initial finding in the posterior lower lobes, following the known disease progression pattern [17]. Future studies focusing on early-stage SSc-ILD patients are needed to validate whether FANO can be identified as an initial manifestation in the posterior lower lobes.

Our longitudinal findings revealed that approximately 70% of cases showed progression, with about half of these developing reticulation. These findings suggest that perivenular fibrosis may form bridging structures, eventually leading to more pronounced fibrotic changes. Furthermore, in cases showing progression, the opacities coalesced to form subpleural curvilinear opacities. While this finding was initially described in asbestosis [35], it has subsequently been reported as characteristic of SSc and polymyositis/dermatomyositis in patients with NSIP [36]. Notably, 78% of FANO demonstrated NSIP on HRCT. Therefore, FANO may capture not only early-stage CT findings of SSc-ILD but also early manifestations of NSIP. Future longitudinal studies with larger cohorts are needed to determine whether FANO corresponds to early-stage CT findings of NSIP and to understand its progression and clinical significance.

Emphysematous changes were more frequently detected pathologically than on CT, including in four never-smokers. This aligns with a previous report showing a higher pathological detection rate, which observed emphysema in 62.5% (10/16) of never-smokers with SSc-ILD [10]. The presence of emphysema in never-smokers suggests mechanisms beyond smoking-related damage. Yamakawa et al. proposed that SSc-related vasculopathy might contribute to emphysematous changes through ischemic injury [10]. However, our study design did not include specific vascular assessments to evaluate this hypothesis. The discrepancy between CT and pathological detection is likely due to CT’s limited spatial resolution for detecting microscopic emphysematous changes.

Our study has several limitations. First, this was a single-institution retrospective study with a small sample size. Additionally, selection bias existed because only patients who could undergo SLB were included. Consequently, mild cases, very severe cases, and cases with severe underlying comorbidities were excluded, potentially not representing the entire SSc-ILD spectrum. Second, the lack of clinical outcome data prevented validation of our imaging findings’ prognostic significance, particularly for UIP-like features identified on HRCT. The clinical utility of these imaging features for predicting disease progression or guiding treatment decisions remains to be established in future studies. Third, we used several types of HRCT scanners, which may have affected the interpretation of subtle CT findings. Finally, SLB specimens were predominantly obtained from the lingula and middle/lower lobes, with no specimens from the upper lobes, where FANO was most frequently observed on CT. However, based on the identical imaging characteristics, we believe that FANO in the upper lobes represents the same pathologic substrate as that confirmed histologically in the lingula and middle/lower lobes.

In conclusion, our study confirms NSIP as the predominant pattern in SSc-ILD, while irregular reticulation with traction bronchiolectasis indicates UIP-like fibrotic features that may have prognostic implications. We newly describe FANO, predominantly observed in the anterolateral upper lobes, as a potential early-stage CT finding corresponding to perivenular fibrosis and peribronchiolar metaplasia. These findings enhance understanding of SSc-ILD pathophysiology and may guide both prognostic assessment and treatment optimization.

## Supplementary Information

Below is the link to the electronic supplementary material.

Supplementary Material 1
