# Supplementary material for: Comprehensive radiologic-pathologic correlation in systemic sclerosis-associated interstitial lung disease: identification of an early-stage CT findings
Source: Jpn J Radiol. 2025 Dec 18;44(4):673–84. doi: 10.1007/s11604-025-01922-2 (PMC13038689; doi:10.1007/s11604-025-01922-2)
Supplement: Supplementary file 1 — Supplementary Material 1 [file 11604_2025_1922_MOESM1_ESM.docx]

**Supplemental Table 1**: Interobserver agreement for whole-lung HRCT findings

| CT findings | Kappa value | Agreement (%) |
| --- | --- | --- |
| Ground-glass opacity | NC* | 100 |
| Consolidation | 0.70 | 89.3 |
| Reticulation | NC* | 100 |
| Centrilobular nodules | 0.56 | 78.6 |
| Traction bronchiectasis | NC* | 100 |
| Emphysema | 0.86 | 92.9 |
| Honeycombing | 0.65 | 96.4 |
| FANO | 0.75 | 89.3 |

*Kappa value could not be calculated because all cases showed positive findings.

*FANO* faint amorphous nodular opacity, *NC* not calculable

**Supplemental Table 2**: Interobserver agreement for biopsy site HRCT findings

| CT findings | Kappa value | Agreement (%) |
| --- | --- | --- |
| Ground-glass opacity | 0.63 | 92.4 |
| Consolidation | 0.67 | 93.7 |
| Irregular reticulation with traction bronchiolectasis | 0.70 | 84.8 |
| Fine reticulation with traction bronchiolectasis | 0.64 | 87.3 |
| Fine reticulation without traction bronchiolectasis | 0.79 | 98.7 |
| Emphysema | 0.73 | 91.1 |
| FANO | 0.59 | 92.4 |

*FANO* faint amorphous nodular opacity

**Supplemental Fig. 1**


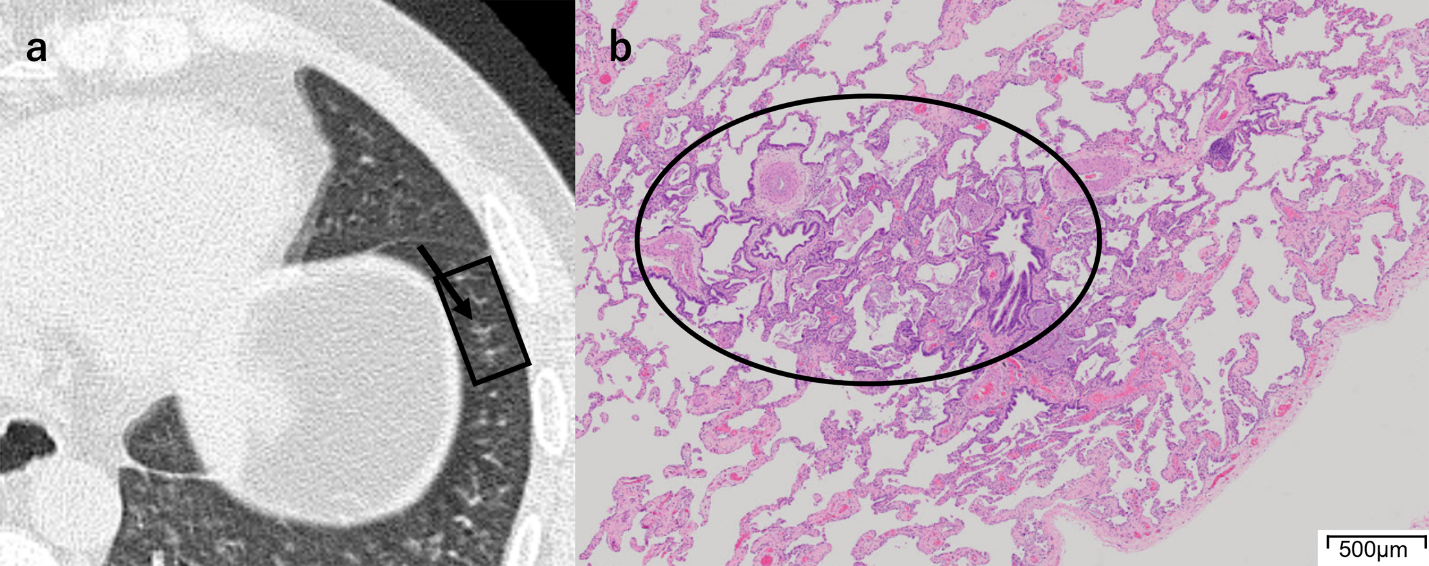


A 39-year-old man with FANO. a Unenhanced axial chest HRCT shows small, faint nodules superimposed on amorphous ground-glass opacity in the left lower lobe (arrow). The SLB resection site is indicated by a black square. b The SLB specimen shows peribronchiolar metaplasia with mucostasis (circle), with diffuse alveolar septal fibrosis in the background. *FANO* faint amorphous nodular opacity, *HRCT* high-resolution CT, *SLB* surgical lung biopsy

**Supplemental Fig. 2**


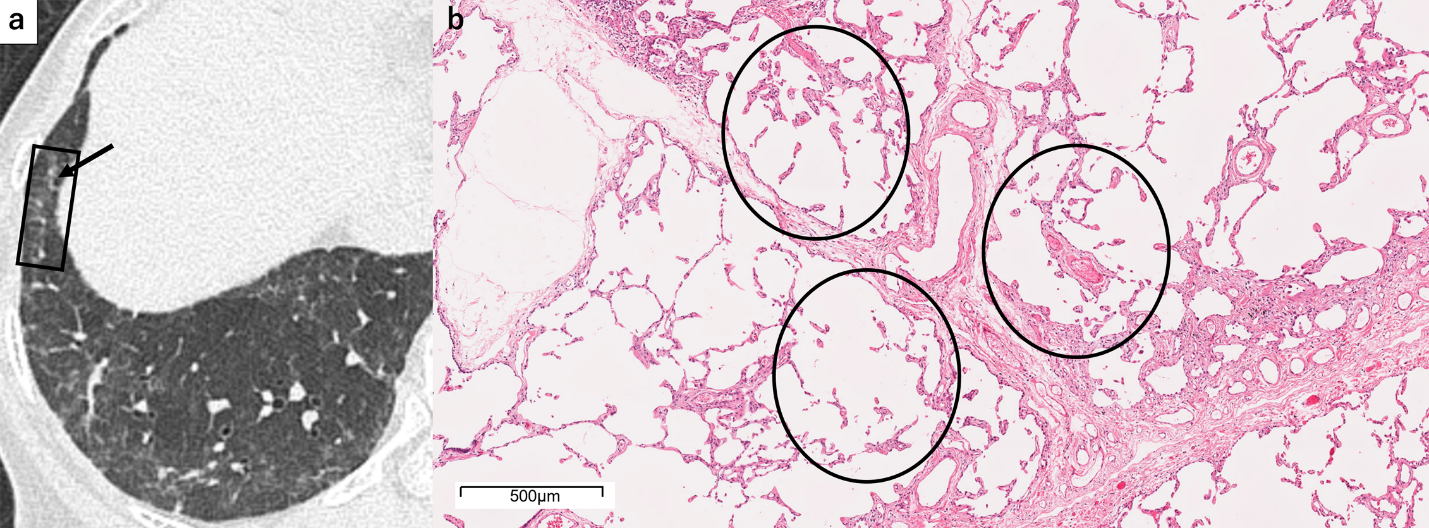


A 70-year-old never-smoking woman with emphysematous change. **a** Unenhanced axial chest HRCT shows fine reticulation with traction bronchiolectasis (arrow), accompanied by surrounding ground-glass opacity; however, emphysema is not apparent. SLB resection site indicated by a black square. **b** The SLB specimen shows fragmented alveolar septa (circles), indicating emphysematous change. *HRCT* high-resolution computed tomography, *SLB* surgical lung biopsy
